# Spatio-temporal evolution and mechanism of regional innovation efficiency: Evidence from Yangtze River Delta Urban Agglomeration of China

**DOI:** 10.1371/journal.pone.0253598

**Published:** 2021-07-01

**Authors:** Xianzhong Cao, Bo Chen, Yuefang Si, Senlin Hu, Gang Zeng

**Affiliations:** Center for Modern Chinese City Studies, Institute of Urban Development, School of Urban & Regional Science, East China Normal University, Shanghai, China; Institute of Geographic Sciences and Natural Resources Research (IGSNRR), Chinese Academy of Sciences (CAS), CHINA

## Abstract

Regional innovation is an important research topic in economic geography, the spatio-temporal evolution and mechanism of regional innovation efficiency have recently become a hot for economic geographers. From the perspective of input and output efficiency, this paper constructs evaluation indicator of regional innovation, with the help of Constant Returns to Scale (CRS) and Variable Returns to Scale (VRS) models, and Malmquist indicator method of Data Envelopment Analysis (DEA), to analyze regional innovation performance, evolution trend, spatial differentiation, and evolution mechanism of Yangtze River Delta Urban Agglomeration (YRDUA) of China. The results show that: (i) Innovation efficiency of YRDUA is generally low, most of which is less than 80 percent of optimal efficiency; however, it kept rising from 2000 to 2015. (ii) Spatial inequality of regional innovation in YRDUA is significant, with a spatial pattern in the shape of “Z”, composed by Hefei, Nanjing, Shanghai, Hangzhou and Ningbo, innovation efficiency of Shanghai is higher than Zhejiang, Anhui and Jiangsu. (iii) Technology progress is the most important influencing factor, all kinds of changing indicator show a trend of rise, and the total factor productivity is changing significantly. This research can provide theoretical reference for the YRDUA to achieve high-quality integration.

## 1. Introduction

The region of Yangtze River Delta in China has many advantages, such as rich talents, high level of science and technology, developed manufacturing industry, complete industrial chain and supply chain, and great market potential [1]. On December 20, 2020, the Ministry of Science and Technology in China issued the Plan for the Construction and Development of Science and Technology Innovation Community in the Yangtze River Delta, focusing on strengthening regional innovation integration in the Yangtze River Delta, optimizing the regional innovation layout, enhancing the regional collaborative innovation capability, and striving to build a globally influential science and technology innovation community in the Yangtze River Delta. Therefore, it is of great significance to study the innovation efficiency of the Yangtze River Delta Urban Agglomeration (YRDUA), which can provide theoretical basis for constructing a regional innovation community in the YRDUA.

Increasing number of studies have shown that economic growth is the result of the interaction among the production, distribution and use of knowledge. Not only local knowledge, but also knowledge flows across economies play an important role [2, 3]. To improve the innovation efficiency is one of the main tasks for regional development. Innovation efficiency refers to the ability of innovation actors in a region to transform innovation input into innovation output in the process of innovation activities, which is divided into narrow sense and broad sense. In the narrow sense, innovation efficiency refers to the ability of the innovator to put new products into the market [4]. In the broad sense, innovation efficiency includes the technical innovation ability of new products and the ability to put them on the market [5]. Guan et al. [6] evaluates the innovation efficiency of cities and countries based on USPTO patent data, they believe that the country’s centrality and structural holes are high, and that the centrality and structural holes of cities have a significant positive effect on innovation efficiency. Frenz and Ietto-Gillies [7] based on the UK community case study found that two different knowledge sources, independent Research and Development and purchase of intellectual property rights, had no significant impact on the firm, but had a positive effect on the efficiency of external firm innovation networks. From the methodology of innovation efficiency, data envelopment analysis [8, 9], negative binomial fixed effect regression model and structural equation model [6], component regression method [10], neural network analysis method [11] and system dynamics method [12] have widely used in the evaluation and prediction of innovation efficiency. Although many scholars have studied innovation efficiency, there are still disputes about the index system construction, spatial differences and influencing factors of innovation efficiency. Some scholars believe that innovation efficiency is innovation ability and can be measured through innovation output, such as patents and papers, the main factors influencing innovation efficiency differences are innovation investment funds and personnel. However, some scholars believe that innovation efficiency should be measured by the ratio of input-output, and the main factor influencing innovation efficiency difference is market demand, etc.

Among all the countries, China has put forward the strategy of innovation-driven development, and innovation plays an irreplaceable role in regional development [2, 13] YRDUA is the sixth largest urban agglomeration in the world, but there are great differences in internal regional development. How to promote innovation-driven development of YRDUA is a realistic problem that cannot be ignored. Before answering this question, many scholars believe that the status, spatial differences and reasons of innovation efficiency of YRDUA should be clarified first [14]. Therefore, this study takes the core cities of YRDUA as the research area. We construct an evaluation index system from the perspective of input and output, and deeply analyze the spatial and temporal evolution characteristics, spatial differences and influencing factors of urban innovation efficiency, which is of great value to the innovation-driven economic development of YRDUA and the regional innovation theory of economic geography.

The remainder of this paper is organized as follows. The next section introduces the indicator system of innovation efficiency. Section 3 describes the data and empirical variables used in analysing the innovation efficiency in YRDUA. With the help of DEA, Section 4 analyses the spatio-temporal evolution of innovation efficiency of 26 cities in YRDUA. Based on malmquist-DEA model, Section 5 discusses the mechanism of regional innovation efficiency. The last section concludes the paper.

## 2. Indicator system of innovation efficiency

The relationship between innovation input and output is expressed as knowledge production function in economics literature [15, 16], there is a consensus on the indicators of innovation input, including the input of Research and Development (R&D) personnel and funding, which are actually frequently referred to in many studies [17]. However, this study believes that R&D personnel with full time input can effectively reflect the input of innovation resources. Therefore, the indicator of innovation input is defined as R&D personnel with full time input (10,000 persons per year) and R&D fund (100 million yuan).

The indicator of innovation output is indeed controversial in academic circle. The quantity of patents and share of new product sales are adopted as indicators of innovation output by Crepon [18]. Some studies find that patent quantity can only partially represent innovation output, while the number of papers published, the balance of technical revenue and expenditure, and the quantity of new products are all variables in innovation output [19]. Hagedron et al. [20] find that the number of patents, the reference rate of patents, and the quantity of new products can indicate the enterprise’s performance in output. Labor productivity and productivity of innovative products are two effective indicators used to measure the enterprise’s performance of output [21]. To sum up, the number of patents, technical papers, the ratio of new product output are chosed to reveal innovation output in this paper. Therefore, innovation input and output can be reflected totally by five indicators (see Table 1). According to the principle of fewer indicators preferred about input-output efficiency, generally, the number of input indicators and output indicators should be no more than the amount of 1/3 Decision Making Units [22]. In this paper, the input-output efficiency measurement in the sampled 26 cities of YRDUA is analyzed, no more than five indicators of input and output will be referred to, and the indicator system shown in Table 1 is qualified.

**Table 1:** The evaluation indicator of innovation efficiency.

Indicator type	Level-I indicator	Level-II indicator
Innovation input	Personnel input	R&D personnel with full time input
	Fund input	R&D fund
Innovation output	Patent output	The number of patents
	Paper output	The number of scientific papers
	New product output	Rate of new product output value

## 3. Methods and data

### 3.1 Constant Returns to Scale (CRS) and Variable Returns to Scale (VRS) models

Data Envelopment Analysis (DEA), which is a non-parametric statistical method created by Charnes in 1978, has become a frequently-adopted method to measure innovation efficiency. Compared with the other evaluation methods, DEA has more advantages, such as the non-necessity to pre-determine the comparability and weight of indicators, and the capacity to provide information to help with identification of low-efficient steps, thus it is convincing in evaluating national or regional innovation efficiency. To evaluate efficiency of innovation input and output in M cities, based on the hypothesis that evaluation indicator system is divided into K input indicators, L output indicators, x_mk_ (x_mk_>0) represents the input of k resource in m city, and y_ml_ (y_ml_>0) indicates the input of l resource in m city. For m (m = 1, 2,… M) city, θ (0<θ≤1) is the comprehensive efficiency indicator of innovation resource input and output (comprehensive efficiency); ε is non-Archimedean infinitesimal number; *λ*_*m*_ (*λ*_*m*_≥0) is variable of weight, to estimate the returns to scale of innovation resources input in cities; s^-^ (s^-^≥0) is slack variable to indicate the amount of innovation resources input that should be deducted to achieve effective DEA; s^+^ (s^+^≥0) is the surplus variable to display the innovation resources output that should be increased to achieve effective DEA. The formulas are as follows:

$$\left\{\begin{array}{c} min\left(\theta -\varepsilon \left(\sum_{k=1}^{K}s^{-}+\sum_{l=1}^{L}s^{+}\right)\right)\\ s.t.\sum_{m=1}^{M}x_{mk}\lambda_{m}+s^{-}=\theta x_{k}^{m}\;k=1,2,\cdots ,K\\ \sum_{m=1}^{M}y_{ml}\lambda_{m}-s^{+}=y_{l}^{m}\;l=1,2,\cdots ,L\\ \lambda_{m}\geq 0\;m=1,2,\cdots ,M \end{array}\right.$$

The formula above is DEA model based on CRS. If optimal solution *θ*_*m*_ = 1 it indicates that the *m* city’s innovation resources run in optimal production frontier, and its innovation output reaches optimal comprehensive efficiency (CE); *θ*_*m*_ < 1, it demonstrates that the *m* city’s innovation resources input is invalid; the closer the value of *θ*_*m*_ to 1, the more efficient the *m* city’s efficiency of innovation resources input-output, and vice versa.

$\sum_{m\;=\;1}^{M}\lambda_{m}\;=\;1\;$ is introduced in the formula which will be changed into DEA model based on VRS. According to VRS model, the comprehensive efficiency can be decomposed into the product of pure technical efficiency (PTE) and scale efficiency (SE), namely *θ*_*m*_ = *θ*_*TE*_ × *θ*_*SE*_. Efficiency indicator *θ*_*m*_ on the basis of VRS model is the comprehensive efficiency of *m* city’s innovation resources input-output, *θ*_*TE*_ is corresponding city agglomeration’s PTE, 0 < *θ*_*TE*_ ≤1, *θ*_*TE*_ ≥ *θ*_m_; Scale efficiency denoted by *θ*_*SE*_, 0 < *θ*_SE_ ≤ 1*,θ*_SE_ ≥ *θ*_m_. The smaller gap between *θ*_*TE*_, *θ*_*SE*_ and 1, the higher efficiency of PTE and SE of innovation resource input-output. *θ*_*TE*_ = 1 and *θ*_*SE*_ = 1 respectively mean optimal PTE or optimal SE of city innovation resources input-output.

In this paper, the efficiency of innovation resources input and output is designed as CE, covering configuration and utilization efficiency of innovation resources; PTE refers to the production efficiency driven by technical progress; SE refers to the gap between existing scale and optimum scale of innovation resources.

### 3.2 Malmquist-DEA model

Malmquist indicator was originally proposed by Malmquist as a consumption indicator and then applied as indicator to measure productivity change by Caves. If Total Factor Productivity (TFP) growth indicator >1, it means positive total factor productivity growth, if total factor productivity growth indicator <1, it means negative total factor productivity growth. Malmquist indicator mainly measures total factor productivity growth, efficiency change and technical progress rate, and in this study it is used to measure the change of efficiency of innovation resources input-output of YRDUA during 2000–2015.

### 3.3 Factor analysis rating method

According to the essence of efficiency evaluation, the innovation resources are input, the results are output, the output is divided by the input. Therefore, in this study, Factor Analysis Rating method (FAR) is adopted for measurement, and comparative analysis with the results of DEA. Efficiency measure formula:

Ei=CiTi

Where in the formula, *E*_*i*_ means technical innovation efficiency in *i* year; *C*_*i*_ is the total composite value of technical innovation output in *i* year, and $C_{i}\;=\;\sum_{j\;=\;1}^{3}\lambda_{j}C_{ij}$ (j = 1, 2, 3) is the value of three technical innovation output in the *i* year. *λ*_*j*_ (j = 1, 2, 3) is the indicator weight of three technical innovation output. *T*_*i*_ is the total value of technical innovation input in *i* year, $T_{i}\;=\;\sum_{j\;=\;1}^{2}\theta_{j}T_{ij}$ (j = 1, 2) is the total value of two technical innovation input in *i* year. *θ*_*j*_ (j = 1, 2) is the indicator weight of two technical innovation input.

In addition, range method is adopted for data processing, and the data are standardized according to the following steps:

Forward indicator:
$$W_{n}\;=\;\frac{X_{n}-X_{min}}{X_{max}-X_{min}}$$
Adverse indicator:
$$W_{n}\;=\;\frac{X_{max}-X_{n}}{X_{max}-X_{min}}$$

The weight of each indicator determined on the basis of factor analysis rating method is respectively: R&D personnel with full time input (0.211), R&D expenditures (0.296), the number of patents (0.493), the number of scientific papers (0.519), and rate of new product output value (0.481).

### 3.4 The sample cities and data source

The sample cities of this study contain 26 cities in YRDUA, namely Shanghai, Hangzhou, Ningbo, Jiaxing, Huzhou, Shaoxing, Zhoushan, Taizhou (in Zhejiang province), Jinhua, Nanjing, Yangzhou, Zhenjiang, Changzhou, Wuxi, Suzhou, Yangzhou, Taizhou (in Jiangsu province), Nantong, Yancheng, Hefei, Wuhu, Maanshan, Tongling, Anqing, Chuzhou, Chizhou and Xuancheng. Due to the difficulty in data collection, after the vertical comparative analysis of data during 2000–2015, the cross-section data of 2000, 2005, 2010 and 2015 are collected and analyzed. In view of the characteristics of R&D activities, and it exits the time-lag effect from input to output, the co-integration analysis shows that the maximum lagged time period is one year, therefore, the innovation output indicators are data of t year, and input indicators are data of t-1 year.

The basic data are mainly from the sixty cities’ Municipal Statistics Bulletin (1999–2016) and their statistical yearbook (2001–2016), Economic Information Committee websites, relevant news reports and existing related research literatures. The scientific papers are from Chongqing VIP Chinese science and technology periodical database (http://www.cqvip.com/) and Web of Knowledge database (http://apps.webofknowledge.com/UA), and the patent data are from the State Intellectual Property Office (SIPO).

## 4. Empirical results

Software DEAP 2.1 is employed to calculate the values of CE, PTE and SE (see Table 2).

**Table 2:** The efficiency of innovation resources input-output of YRDUA during 2000–2015.

City	2000			2005			2010			2015		
	CE	PTE	SE	CE	PTE	SE	CE	PTE	SE	CE	PTE	SE
Shanghai	0.589	1.000	0.589	1.000	1.000	1.000	1.000	1.000	1.000	1.000	1.000	1.000
Hangzhou	0.629	1.000	0.629	0.328	1.000	0.328	0.700	0.702	0.997	1.000	1.000	1.000
Ningbo	0.718	0.932	0.771	0.345	0.673	0.513	1.000	1.000	1.000	0.996	1.000	0.996
Huzhou	0.848	0.934	0.908	0.327	1.000	0.327	0.663	1.000	0.663	0.933	1.000	0.933
Jiaxing	0.316	0.346	0.915	0.167	0.794	0.211	0.236	0.248	0.952	0.282	0.311	0.909
Shaoxing	0.274	0.473	0.578	0.140	0.559	0.250	0.579	0.756	0.766	0.359	0.360	0.997
Zhoushan	0.631	1.000	0.631	0.402	0.478	0.842	1.000	1.000	1.000	1.000	1.000	1.000
Taizhou (Zhejiang)	0.844	1.000	0.844	0.241	0.912	0.264	0.707	0.781	0.906	1.000	1.000	1.000
Nanjing	0.649	1.000	0.649	1.000	1.000	1.000	1.000	1.000	1.000	1.000	1.000	1.000
Suzhou	0.534	0.882	0.605	0.774	0.795	0.974	0.192	0.192	0.999	0.248	0.251	0.989
Wuxi	0.535	0.867	0.618	0.479	0.499	0.959	0.754	0.766	0.985	0.334	0.335	0.999
Changzhou	0.441	0.487	0.905	0.583	0.638	0.914	0.344	0.359	0.959	0.460	0.477	0.965
Zhenjiang	0.569	0.666	0.854	0.185	0.294	0.628	0.258	0.266	0.971	0.494	0.525	0.942
Yangzhou	0.325	0.434	0.748	0.237	0.245	0.970	0.347	1.000	0.347	0.447	0.452	0.989
Taizhou (Jiangsu)	0.265	0.314	0.844	0.144	0.152	0.947	0.146	0.174	0.839	0.162	0.211	0.770
Nantong	1.000	1.000	1.000	0.199	0.228	0.874	0.211	0.217	0.973	0.225	0.240	0.937
Jinhua	0.087	0.291	0.300	0.257	1.000	0.257	0.364	0.527	0.691	0.732	0.750	0.976
Yancheng	0.222	0.672	0.330	0.174	0.224	0.774	0.471	0.714	0.659	0.711	0.754	0.942
Hefei	0.331	0.823	0.402	0.632	0.766	0.826	1.000	1.000	1.000	1.000	1.000	1.000
Wuhu	0.126	0.202	0.622	0.833	0.890	0.936	0.395	0.701	0.563	0.374	0.430	0.868
Maanshan	0.224	0.318	0.703	0.822	0.874	0.940	1.000	1.000	1.000	0.707	0.957	0.739
Tongling	0.302	0.618	0.489	0.539	1.000	0.539	1.000	1.000	1.000	1.000	1.000	1.000
Anqing	0.317	0.750	0.422	1.000	1.000	1.000	0.999	1.000	0.999	1.000	1.000	1.000
Chuzhou	0.100	0.168	0.595	0.656	1.000	0.656	0.316	0.330	0.958	0.362	0.550	0.658
Chizhou	1.000	1.000	1.000	1.000	1.000	1.000	1.000	1.000	1.000	1.000	1.000	1.000
Xuancheng	0.169	0.292	0.579	0.251	0.335	0.750	0.376	0.394	0.955	0.521	0.907	0.574
Average	0.463	0.672	0.674	0.489	0.706	0.718	0.618	0.697	0.892	0.667	0.712	0.930

Note: CE refers to Comprehensive Efficiency, PTE refers to Pure Technical Efficiency, SE refers to Scale Efficiency.

### 4.1 The efficiency of innovation resources input-output of YRDUA is generally low but gradually rising

The CE of innovation resources input-output of YRDUA is generally low but gradually rising. The values of CE in 2000, 2005, 2010 and 2015 are 0.463, 0.489, 0.618 and 0.667 respectively, which is less than 70 percent of the optimal efficiency. The average value of CE has increased by 0.204 from 2000 to 2015. The CE of Nantong and Chizhou reached the optimal level in 2000, and Shanghai, Nanjing, Anqing and Chizhou reached the optimal level in 2005, and Shanghai, Ningbo, Zhoushan, Nanjing, Hefei, Maanshan, Tongling and Chizhou reached the optimal level in 2010, and Shanghai, Hangzhou, Zhoushan, Taizhou (Zhejiang), Nanjing, Hefei, Tongling, Anqing and Chizhou reached the optimal level in 2015. In addition, the values of CE of eighteen cities are below 60 percent of the optimal level in 2000, which is 69.23 percent of all cities; The values of CE of four cities are above 80 percent of the optimal level in 2000, which is 15.38 percent of all cities; The values of CE of four cities are between 60 percent and 80 percent of the optimal level in 2000, which is 15.38 percent of all cities. However, in 2015, the values of CE of twelve cities are below 60 percent of the optimal level, which is 46.15 percent of all cities; The values of CE of eleven cities are above 80 percent of the optimal level, which is 42.31 percent of all cities; The values of CE of three cities are between 60 percent and 80 percent of the optimal level, which is 11.54 percent of all cities. Overall, the efficiency of innovation resources input-output of YRDUA is generally low and failed to reach the optimal level during 2000–2015, however, it gradually increased. This study finds that if cities with higher or lower economic development level and cities, the cities’ innovation efficiency will be higher, however, the innovation efficiency of cities with medium development level will be lower, the cause is that innovative resources are abundant and higher innovation output and utilization rate in cities with higher level of economic development, and innovation resources are scarce in cities with lower level of economic development, but innovation output and utilization rate are higher than the cities with medium level of economic development. This is also consistent with the existing research, such as Teng and Fang [23].

The PTE of innovation resources input-output of YRDUA is generally higher than the CE and gradually rising. The PTE in 2000, 2005, 2010 and 2015 are 0.672, 0.706, 0.697 and 0.712 respectively, which all reached 60 percent of the optimal efficiency. Compared to the CE, the PTE is generally higher, despite slight decline during 2005–2010, it rose by 0.040 from 2000 to 2015. The PTE of 7 cities have reached the optimal level in 2000, and the number rises to 11 in 2015. In 2000, the values of PTE of 12 cities are above 80 percent of the optimal level, which is 46.15 percent of all cities; The values of PTE of ten cities are below 60 percent of the optimal level, which is 38.46 percent of all cities; The values of PTE of 4 cities are between 60 percent and 80 percent of the optimal level, which is 15.39 percent of all cities. In 2015, the values of PTE of 13 cities are above 80 percent of the optimal level, which is 50 percent of all cities; The values of PTE of 11 cities are below 60 percent of the optimal level, which is 42.31 percent of all cities; Only 2 cities are between 60 percent and 80 percent of the optimal level, which is 7.69 percent of all cities. The PTE of innovation resources input-output of YRDUA is greatly improved during 2000–2015, which also shows that technical level has made great progress.

From the point of view of SE, it is significantly higher than CE and PTE during the same period. SE in 2000, 2005, 2010 and 2015 are respectively 0.674, 0.718, 0.892 and 0.930, has reached above 60 percent of the optimal level, and it increased by 0.256 from 2000 to 2015. In 2000, SE of two cities reached the optimal level, and nine cities did so in 2015. Moreover, eight cities’ SE are above 80 percent of the optimal, and nine cities are below 60 percent of the optimal in 2000; Twenty-two cities’ SE are above 80 percent, only Xuancheng is below 60 percent. Overall, SE of innovation resources input-output of YRDUA is generally high and increasing, and is significantly higher than CE and PTE.

### 4.2 Comparison of innovation efficiency in YRDUA

The efficiency of innovation resources input-output of the core cities of YRDUA during 2000 to 2015 is overall high, such as Shanghai, Hangzhou, Ningbo, Nanjing, Hefei. CE of Shanghai, Hangzhou, Zhoushan, Taizhou (Zhejiang), Nanjing, Hefei, Tongling, Anqing and Chizhou has reached the optimal level in 2015, but Suzhou is only 0.248, and the PTE and SE are respectively 0.251 and 0.989. The results demonstrate no fault in Suzhou innovation resources input or output, and it is the poor matching between innovation resources scale and input-output, which causes low CE of innovation resources input-output in Suzhou. Deap 2.1 calculation results indicate the output does not increase correspondingly with the input of innovation increases in Suzhou, and it implys that local CE can be enhanced by reducing the scale of innovation resources input or increasing innovation output, even in new product output. In addition, although the CE of innovation resources input-output in Jiaxing, Shaoxing, Wuxi, Changzhou, Zhenjiang, Yangzhou, Maanshan, Chuzhou and Xuancheng is higher than Suzhou, their returns to innovation resources input scale gradually decline as Suzhou’s, so do those in Jinhua, Yancheng and Wuhu, however, they fails to reach the optimal, due to redundant R&D personnel and expenditure, and innovation output is poor, especially in patents output. For the remaining two cities, such as Ningbo and Huzhou, the returns to innovation resources input scale increase gradually, but CE fail to reach the optimal level mainly due to input redundancy and output deficiency, especially the output value of new products that needs further significantly improving, Huzhou is weak in output of scientific papers, which can be improved by increasing innovation resources scale and encouraging development of new products.

As is clearly revealed by Table 2, CE of innovation resources input-output of the cities of Shanghai municipality and Zhejiang province are obviously superior to the cities of Jiangsu and Anhui province, the gap among the cities of Zhejiang and Anhui province is small, while that among the cities in Jiangsu province is large. In order to more clearly reveal the efficiency of innovation resources input-output of different province in YRDUA, this research estimated CE of Shanghai, Jiangsu, Zhejiang and Anhui in 2015 (see Table 3), the CE of Shanghai, Jiangsu, Zhejiang and Anhui are respectively 1.000, 0.653, 0.768 and 0.726, only Shanghai’s PTE reached the optimal level in 2015, which indicates that the scale of innovation resources input is critical for increase of CE, further study manifests decline of returns to scale, it will be necessity to reasonably increase the scale of innovation resources output and improve the CE in Jiangsu, Zhejiang, and Anhui.

**Table 3:** The efficiency of innovation resources input-output of province level of YRDUA in 2015.

Region	Comprehensive efficiency	Pure technical efficiency	Scale efficiency
Shanghai	1.000	1.000	1.000
Jiangsu	0.653	0.772	0.846
Zhejiang	0.768	0.872	0.881
Anhui	0.726	0.835	0.869

According to longitudinal comparison of the efficiency of innovation resources input-output in the cities of YRDUA, the spatial differentiation among the cities reduces gradually from 2000 to 2015, but the internal spatial pattern changes greatly.

The value of CE of innovation resources input-output of YRDUA rises in Shanghai, Nanjing, Suzhou, Changzhou, Jinhua, Hefei, Wuhu, Maanshan, Tongling, Anqing, Chuzhou and Xuancheng during 2000–2005. Shanghai, Nanjing and Anqing exactly speaking has reached the optimal level, Wuhu and Maanshan are above 80 percent of the optimal, but the other cities stay low efficiency, Shanghai, Nanjing, Jinhua, Hefei, Wuhu, Maanshan, Anqing and Chuzhou experienced major changes. As is demonstrated by further analysis, the great fluctuation of Shanghai’s and Nanjing’s CE of innovation resources input-output mainly lies in the decreasing returns to scale and corresponding reduction of innovation resources, while Anqing reached the optimal level lies in reasonable control of the innovation resources input and output. The CE decreases in Hangzhou, Ningbo, Huzhou, Jiaxing, Shaoxing, Zhoushan, Taizhou (Zhejiang), Wuxi, Zhenjiang, Yangzhou, Taizhou (Jiangsu), Nantong and Yancheng, which is mainly caused by excessive R&D expenditures and low output value of new products and patents. Chizhou has always been the optimal level from 2000 to 2005.

There are great changes of comprehensive efficiency of innovation resources input of YRDUA during 2005–2010. Eight cities has achieved the optional level, such as Shanghai, Ningbo, Zhoushan, Nanjing, Hefei, Maanshan, Tongling and Chizhou. The cities with CE rose include Hangzhou, Ningbo, Huzhou, Jiaxing, Shaoxing, Zhoushan, Taizhou (Zhejiang), Wuxi, Zhenjiang, Yangzhou, Taizhou (Jiangsu), Nantong, Jinhua, Yancheng, Hefei, Maanshan, Tongling and Xuancheng, among which Ningbo, Zhoushan, Hefei, Maanshan and Tongling are at a higher level, low in the remaining cities, and most fluctuant in Ningbo and Zhoushan, from 0.345 and 0.402 to the optimal level during 2005 to 2010. According to further observation, the course of innovation resources input and output in Ningbo and Zhoushan in 2005 was bothered by serious redundancy of R&D expenditure and the lack of innovative talents, and low output value of new products and scientific papers, which demonstrates reasonable adjustment of innovation resources input in Ningbo and Zhoushan over the five years for optimization; The cities with CE declined include Suzhou, Changzhou, Wuhu, Anqing and Chuzhou, and decreased most seriously in Suzhou, and SE of Suzhou is 0.999, but the PTE is lower, which shows that technological progress has not been fully played on the process of innovation resources input-output, and the transfer and transformation of technological achievements of Suzhou need to be strengthened. The PTE in Anqing is 1.000, which manifests the major cause of CE decline by improper scale of innovation resources input, the necessity to appropriately reduce the scale of innovation resources, and the cause of drop of CE of innovation resources in other cities by excessive R&D personnel and expenditure as well as severe shortage of scientific papers output. Shanghai, Nanjing, Chizhou have always been the optimal level from 2005 to 2010.

From 2010 to 2015, the CE of innovation resources input and output of YRDUA changed little overall and rose in most of the cities. It achieved the optimal level in Shanghai, Hangzhou, Zhoushan, Taizhou (Zhejiang), Hefei, Tongling, Anqing and Chizhou, declined in Ningbo, Shaoxing, Wuxi, Wuhu and Maanshan, increased to different extents in the rest cities and reached relatively higher level in Hangzhou, Huzhou, Taizhou (Zhejiang) and Anqing, but lower in the other cities. Further decline in Shaoxing, Wuxi, Wuhu and Maanshan because of improper scale of innovation resources input, and remained high in Ningbo in spite of slight decline. In addition, it’s worth noting that the CE of innovation resources input in Hangzhou, Taizhou (Zhejiang) and Anqing over five years rose fastly and reached the optimal level in 2015, after in-depth analysis, it is found that R&D personnel and expenditures were reasonably controlled in these cities, and the number of scientific papers and invention patents grew in leaps and bounds.

### 4.3 The efficiency value shows little difference with FAR method and DEA method

The data are processed with the method of Factor Analysis Rating (FAR), in order to achieve more comparable results, the values of efficiency are normalized (see Table 4).

**Table 4:** The difference of efficiency between FAR and DEA method in 2015.

Province/city	FAR efficiency	DEA efficiency	Province/city	FAW efficiency	DEA efficiency
Shanghai	0.923	1.000	Zhenjiang	0.379	0.494
Jiangsu	0.609	0.653	Yangzhou	0.324	0.447
Zhejiang	0.770	0.768	Taizhou (Jiangsu)	0.124	0.162
Anhui	0.687	0.726	Nantong	0.205	0.225
Hangzhou	0.859	1.000	Jinhua	0.553	0.732
Ningbo	0.828	1.000	Yancheng	0.623	0.711
Huzhou	0.780	0.996	Hefei	0.780	1.000
Jiaxing	0.696	0.933	Wuhu	0.291	0.374
Shaoxing	0.208	0.282	Maanshan	0.651	0.707
Zhoushan	0.002	0.359	Tongling	0.799	1.000
Taizhou (Zhejiang)	0.804	1.000	Anqing	0.778	1.000
Nanjing	1.000	1.000	Chuzhou	0.227	0.362
Suzhou	0.850	1.000	Chizhou	0.801	1.000
Wuxi	0.231	0.248	Xuancheng	0.438	0.521
Changzhou	0.338	0.334	—	—	—

According to the data in Table 4, from the perspective of province level, Shanghai’s efficiency of innovation resources input-output is higher than Zhejiang’s, Anhui’s and Jiangsu’s,. Comparatively speaking, the FAR-based value of efficiency is normalized, and DEA-based of efficiency is just a relative value, hence they are different, however, the FAR-based value of efficiency and FAR-based value of efficiency are consistent with the trend of evolution. Approximately speaking, the values of each province’s or city’s efficiency calculated based on the two methods match well with each other. Taking Shanghai as an example, its efficiency based on FAR is 0.923, while that based on DEA is 1.000, both high relatively. However, the two values of Zhoushan’s vary significantly, which is due to minimum values of Zhoushan’s indicators in the process of data standardization. Therefore, the FAW-based value verifies the DEA-based result and demonstrates the effectiveness of DEA method in the calculation of efficiency of innovation resources input-output.

## 5. The evolution mechanism of regional innovation performance

Based on the Malmquist-DEA model, malmquist indicators during 2000 to 2015 about innovation resources input and output of 26 cities in YRDUA are estimated with the software of DEAP 2.1 (see Table 5). The indicator of CE fluctuation(1.110), technical progress fluctuation (1.001), PTE fluctuation (1.047), SE fluctuation (1.060) and total factor productivity fluctuation (1.111) are all greater than 1.000, which indicates that the significant increase of total factor productivity of innovation resources of YRDUA is right the result of technical progress, and CE improvement is mainly promoted by PTE increase, the scale advantages of innovation resources are slightly made good use, and technology progress plays a pivotal role in improvement of efficiency of innovation resources input and output. From different cities, this research found that the total factor productivity fluctuation of eight cities in Anhui province and Zhoushan and Wuxi are all less than 1.000, the other cities are more than 1.000, in the other word, technical progress has little effect on the improvement of these cities’ innovation capability and the utilization rate of innovation resources is not high. The spatial evolution process of total factor productivity fluctuation in YRDUA from 2000 to 2015, which has shaped a “Z” with Shanghai as the core and Hefei, Nanjing, Hangzhou and Ningbo as the nodes of axis, which is also the motivation for the spatio-temporal evolution of the efficiency of innovation resources input-output of 26 cities in YRDUA.

**Table 5:** The Malmquist index of YRDUA during 2000 to 2015.

City	Comprehensive efficiency fluctuation	Technical progress fluctuation	Pure technical efficiency fluctuation	Scale efficiency fluctuation	Total factor productivity fluctuation
Shanghai	1.000	2.119	1.000	1.000	2.119
Hangzhou	1.428	1.742	1.424	1.003	2.488
Ningbo	0.996	1.335	1.000	0.996	1.330
Huzhou	1.407	0.712	1.000	1.407	1.002
Jiaxing	1.195	1.260	1.252	0.954	1.505
Shaoxing	0.619	1.150	0.476	1.302	0.712
Zhoushan	1.000	0.636	1.000	1.000	0.636
Taizhou (Zhejiang)	1.414	1.478	1.281	1.104	2.089
Nanjing	1.000	1.660	1.000	1.000	1.660
Suzhou	1.295	1.451	1.308	0.990	1.878
Wuxi	0.443	1.517	0.437	1.014	0.672
Changzhou	1.336	1.518	1.327	1.007	2.029
Zhenjiang	1.916	1.338	1.977	0.969	2.563
Yangzhou	1.289	1.124	0.452	2.852	1.449
Taizhou (Jiangsu)	1.111	1.418	1.211	0.918	1.576
Nantong	1.065	1.420	1.106	0.963	1.511
Jinhua	2.011	0.706	1.423	1.413	1.419
Yancheng	1.510	0.726	1.056	1.430	1.096
Hefei	1.000	0.872	1.000	1.000	0.872
Wuhu	0.946	0.566	0.614	1.542	0.536
Maanshan	0.707	0.811	0.957	0.739	0.573
Tongling	1.000	0.408	1.000	1.000	0.408
Anqing	1.001	0.764	1.000	1.001	0.764
Chuzhou	1.145	0.688	1.667	0.687	0.788
Chizhou	1.000	0.632	1.000	1.000	0.632
Xuancheng	1.384	0.462	2.301	0.602	0.640
Average	1.110	1.001	1.047	1.060	1.111

## 6. Conclusion and discussion

In this study, the efficiency of innovation resources input-output of the Yangtze River Delta Urban Agglomeration (YRDUA) from 2000 to 2015 is measured with the models of Constant Returns to Scale (CRS) and Variable Returns to Scale (VRS) of Data Envelopment Analysis (DEA), and the results are verified with the method of Factor Analysis Rating method (FAR); in addition, the efficiency fluctuation (2000–2015) is estimated on the basis of Malmquist-DEA model, and the following conclusions are drawn.

First, CE of innovation resources input-output of YRDUA is generally low and inferior to the optimal level, but tends to rise from 2000 to 2015; Pure technical efficiency is generally higher than CE and increasing; Scale efficiency is significantly higher than CE and PTE of the same period.

Second, Shanghai’s efficiency of innovation resources input-output is significantly higher than Zhejiang’s, Anhui’s and Jiangsu’s, which is the lowest; The gap among the different Yangtze River Delta cities’ efficiency of innovation resources input-output gradually reduces, however, the spatial pattern changes a lot; The indexes of fluctuation of efficiencies of YRDUA’s innovation resources input-output are gradually increasing, total factor productivity changes violently, technical progress plays a decisive role in efficiency enhancement of innovation resources input and output. The overall low efficiency of innovation resources input-output is mainly the consequence of decreasing returns to scale of innovation resources input or extravagant R&D expenditures, followed by weak output.

Third, the values of efficiencies based on the method of Factor Analysis Rating are approximate to those based on DEA, which certifies the effectiveness of DEA method in the calculation of efficiency of innovation resources input-output.

Regional innovation is long-term concerns of economic geographers [24–26]. Innovation includes the production of new products, the adoption of new production methods, the development of new markets, the expansion of new sources of supply or new forms of organization, etc, acquiring innovative knowledge is a prerequisite for the implementation of innovation activities [27]. The spatio-temporal evolution of input and output efficiency of regional innovation resources is not only a research topic concerned by economic geographers, but also a realistic problem that policy makers and enterprise managers urgently need to solve. Many scholars have carried out research on innovation efficiency, but the current research mainly discusses the inequality of innovation space, there is a lack of research on innovation efficiency, especially on the spatio-temporal evolution and mechanism of regional innovation efficiency based on DEA, FAR and Malmquist-DEA model, which is discussed by this research, and also drawn some interesting conclusions. However, considering the data availability, the cross-sectional data from four periods are adopted, and hereby it is suggested to consider more periods and further explore the influential factors and motivation of efficiency differentiation of innovation input and output in further studies.

## Supporting information

S1 Data (DOCX)
