# Supplementary material for: Spatio-temporal evolution and mechanism of regional innovation efficiency: Evidence from Yangtze River Delta Urban Agglomeration of China
Source: PLoS One. 2021 Jul 1;16(7):e0253598. doi: 10.1371/journal.pone.0253598 (PMC8248968; doi:10.1371/journal.pone.0253598)
Supplement: S1 Data — (DOCX) [file pone.0253598.s001.docx]

Results from DEAP Version 2.1

Instruction file = 123.ins

Data file = 123.dta

Input orientated Malmquist DEA

DISTANCES SUMMARY

year = 1

firm crs te rel to tech in yr vrs

no. ************************ te

t-1 t t+1

1 0.000 1.000 0.722 1.000

2 0.000 0.700 0.526 0.702

3 0.000 1.000 0.868 1.000

4 0.000 0.663 1.491 1.000

5 0.000 0.236 0.212 0.248

6 0.000 0.579 0.665 0.756

7 0.000 1.000 2.865 1.000

8 0.000 0.707 0.548 0.781

9 0.000 1.000 0.767 1.000

10 0.000 0.192 0.148 0.192

11 0.000 0.754 0.559 0.766

12 0.000 0.344 0.259 0.359

13 0.000 0.258 0.221 0.266

14 0.000 0.347 0.432 1.000

15 0.000 0.146 0.115 0.174

16 0.000 0.211 0.164 0.217

17 0.000 0.364 0.619 0.527

18 0.000 0.471 0.694 0.714

19 0.000 1.000 1.667 1.000

20 0.000 0.395 0.919 0.701

21 0.000 1.000 1.621 1.000

22 0.000 1.000 3.181 1.000

23 0.000 0.999 1.405 1.000

24 0.000 0.316 0.469 0.330

25 0.000 1.000 3.005 1.000

26 0.000 0.376 0.853 0.394

mean 0.000 0.618 0.961 0.697

year = 2

firm crs te rel to tech in yr vrs

no. ************************ te

t-1 t t+1

1 3.240 1.000 0.000 1.000

2 2.278 1.000 0.000 1.000

3 1.543 0.996 0.000 1.000

4 1.063 0.933 0.000 1.000

5 0.402 0.282 0.000 0.311

6 0.545 0.359 0.000 0.360

7 1.157 1.000 0.000 1.000

8 1.691 1.000 0.000 1.000

9 2.112 1.000 0.000 1.000

10 0.403 0.248 0.000 0.251

11 0.570 0.334 0.000 0.335

12 0.799 0.460 0.000 0.477

13 0.757 0.494 0.000 0.525

14 0.703 0.447 0.000 0.452

15 0.257 0.162 0.000 0.211

16 0.351 0.225 0.000 0.240

17 0.620 0.732 0.000 0.750

18 0.552 0.711 0.000 0.754

19 1.269 1.000 0.000 1.000

20 0.279 0.374 0.000 0.430

21 0.753 0.707 0.000 0.957

22 0.529 1.000 0.000 1.000

23 0.820 1.000 0.000 1.000

24 0.254 0.362 0.000 0.550

25 1.200 1.000 0.000 1.000

26 0.252 0.521 0.000 0.907

mean 0.939 0.667 0.000 0.712

[Note that t-1 in year 1 and t+1 in the final year are not defined]

MALMQUIST INDEX SUMMARY

year = 2

firm effch techch pech sech tfpch

|  | 1 | 1.000 | 2.119 | 1.000 | 1.000 | 2.119 |
| --- | --- | --- | --- | --- | --- | --- |
|  | 2 | 1.428 | 1.742 | 1.424 | 1.003 | 2.488 |
|  | 3 | 0.996 | 1.335 | 1.000 | 0.996 | 1.330 |
|  | 4 | 1.407 | 0.712 | 1.000 | 1.407 | 1.002 |
|  | 5 | 1.195 | 1.260 | 1.252 | 0.954 | 1.505 |
|  | 6 | 0.619 | 1.150 | 0.476 | 1.302 | 0.712 |
|  | 7 | 1.000 | 0.636 | 1.000 | 1.000 | 0.636 |
|  | 8 | 1.414 | 1.478 | 1.281 | 1.104 | 2.089 |
|  | 9 | 1.000 | 1.660 | 1.000 | 1.000 | 1.660 |
|  | 10 | 1.295 | 1.451 | 1.308 | 0.990 | 1.878 |
|  | 11 | 0.443 | 1.517 | 0.437 | 1.014 | 0.672 |
|  | 12 | 1.336 | 1.518 | 1.327 | 1.007 | 2.029 |
|  | 13 | 1.916 | 1.338 | 1.977 | 0.969 | 2.563 |
|  | 14 | 1.289 | 1.124 | 0.452 | 2.852 | 1.449 |
|  | 15 | 1.111 | 1.418 | 1.211 | 0.918 | 1.576 |
|  | 16 | 1.065 | 1.420 | 1.106 | 0.963 | 1.511 |
|  | 17 | 2.011 | 0.706 | 1.423 | 1.413 | 1.419 |
|  | 18 | 1.510 | 0.726 | 1.056 | 1.430 | 1.096 |
|  | 19 | 1.000 | 0.872 | 1.000 | 1.000 | 0.872 |
|  | 20 | 0.946 | 0.566 | 0.614 | 1.542 | 0.536 |
|  | 21 | 0.707 | 0.811 | 0.957 | 0.739 | 0.573 |
|  | 22 | 1.000 | 0.408 | 1.000 | 1.000 | 0.408 |
|  | 23 | 1.001 | 0.764 | 1.000 | 1.001 | 0.764 |
|  | 24 | 1.145 | 0.688 | 1.667 | 0.687 | 0.788 |
|  | 25 | 1.000 | 0.632 | 1.000 | 1.000 | 0.632 |
|  | 26 | 1.384 | 0.462 | 2.301 | 0.602 | 0.640 |

mean 1.110 1.001 1.047 1.060 1.111

MALMQUIST INDEX SUMMARY OF ANNUAL MEANS

year effch techch pech sech tfpch

2 1.110 1.001 1.047 1.060 1.111

mean 1.110 1.001 1.047 1.060 1.111

MALMQUIST INDEX SUMMARY OF FIRM MEANS

firm effch techch pech sech tfpch

1 1.000 2.119 1.000 1.000 2.119

2 1.428 1.742 1.424 1.003 2.488

3 0.996 1.335 1.000 0.996 1.330

4 1.407 0.712 1.000 1.407 1.002

5 1.195 1.260 1.252 0.954 1.505

6 0.619 1.150 0.476 1.302 0.712

7 1.000 0.636 1.000 1.000 0.636

8 1.414 1.478 1.281 1.104 2.089

9 1.000 1.660 1.000 1.000 1.660

10 1.295 1.451 1.308 0.990 1.878

11 0.443 1.517 0.437 1.014 0.672

12 1.336 1.518 1.327 1.007 2.029

13 1.916 1.338 1.977 0.969 2.563

14 1.289 1.124 0.452 2.852 1.449

15 1.111 1.418 1.211 0.918 1.576

16 1.065 1.420 1.106 0.963 1.511

17 2.011 0.706 1.423 1.413 1.419

18 1.510 0.726 1.056 1.430 1.096

19 1.000 0.872 1.000 1.000 0.872

20 0.946 0.566 0.614 1.542 0.536

21 0.707 0.811 0.957 0.739 0.573

22 1.000 0.408 1.000 1.000 0.408

23 1.001 0.764 1.000 1.001 0.764

24 1.145 0.688 1.667 0.687 0.788

25 1.000 0.632 1.000 1.000 0.632

26 1.384 0.462 2.301 0.602 0.640

mean 1.110 1.001 1.047 1.060 1.111

[Note that all Malmquist index averages are geometric means]
